# Isoflurane Alters Presynaptic Endoplasmic Reticulum Calcium Dynamics in Wild-Type and Malignant Hyperthermia-Susceptible Rodent Hippocampal Neurons

**DOI:** 10.1523/ENEURO.0114-23.2023

**Published:** 2023-08-28

**Authors:** Vanessa Osman, Iris Speigel, Kishan Patel, Hugh C. Hemmings

**Affiliations:** 1 Department of Pharmacology, Weill Cornell Medical College, New York, NY 10065; 2 Department of Anesthesiology, Weill Cornell Medical College, New York, NY 10065

**Keywords:** anesthesia, calcium, endoplasmic reticulum, exocytosis, isoflurane, malignant hyperthermia, presynaptic, propofol, sevoflurane, synaptic vesicle

## Abstract

Volatile anesthetics reduce excitatory synaptic transmission by both presynaptic and postsynaptic mechanisms which include inhibition of depolarization-evoked increases in presynaptic Ca^2+^ concentration and blockade of postsynaptic excitatory glutamate receptors. The presynaptic sites of action leading to reduced electrically evoked increases in presynaptic Ca^2+^ concentration and Ca^2+^-dependent exocytosis are unknown. Endoplasmic reticulum (ER) of Ca^2+^ release via ryanodine receptor 1 (RyR1) and uptake by SERCA are essential for regulation intracellular Ca^2+^ and are potential targets for anesthetic action. Mutations in sarcoplasmic reticulum (SR) release channels mediate volatile anesthetic-induced malignant hyperthermia (MH), a potentially fatal pharmacogenetic condition characterized by unregulated Ca^2+^ release and muscle hypermetabolism. However, the impact of MH mutations on neuronal function are unknown. We used primary cultures of postnatal hippocampal neurons to analyze volatile anesthetic-induced changes in ER Ca^2+^ dynamics using a genetically encoded ER-targeted fluorescent Ca^2+^ sensor in both rat and mouse wild-type (WT) neurons and in mouse mutant neurons harboring the *RYR1* T4826I MH-susceptibility mutation. The volatile anesthetic isoflurane reduced both baseline and electrical stimulation-evoked increases in ER Ca^2+^ concentration in neurons independent of its depression of presynaptic cytoplasmic Ca^2+^ concentrations. Isoflurane and sevoflurane, but not propofol, depressed depolarization-evoked increases in ER Ca^2+^ concentration significantly more in mouse *RYR1* T4826I mutant neurons than in wild-type neurons. The *RYR1* T4826I mutant neurons also showed markedly greater isoflurane-induced reductions in presynaptic cytosolic Ca^2+^ concentration and synaptic vesicle (SV) exocytosis. These findings implicate RyR1 as a molecular target for the effects of isoflurane on presynaptic Ca^2+^ handling.

## Significance Statement

Despite their essential clinical roles, the molecular and cellular mechanisms of action of general anesthetics are not fully understood. Malignant hyperthermia (MH) is a potentially fatal pharmacogenetic disorder that leads to dysregulation of intracellular Ca^2+^ handling in response to triggering by volatile anesthetics. While research on malignant hyperthermia has focused on skeletal muscle effects, much less is known about its neuronal effects. We identify neuronal endoplasmic reticulum (ER) Ca^2+^ regulation as a novel target for volatile anesthetic action and as a potential target in malignant hyperthermia. While depression of CNS electrical activity *in vivo* by anesthesia has been observed in another model of malignant hyperthermia, our study reveals fundamental presynaptic mechanisms of volatile anesthetics with implications for the development of more selective anesthetics and for prevention and treatment of malignant hyperthermia.

## Introduction

Although volatile anesthetics are essential to modern medicine, a detailed understanding of their cellular and molecular mechanisms of action is incomplete despite 175 years of clinical use. In addition to their major effect of producing an unconsciousness that allows for painful procedures, volatile anesthetics also produce serious cardiovascular and respiratory side effects, and can trigger the rare but potentially fatal pharmacogenetic reaction malignant hyperthermia (MH). Malignant hyperthermia is a hypermetabolic syndrome characterized by excessive intracellular Ca^2+^ release from the sarcoplasmic reticulum (SR) in skeletal muscle leading to hyperthermia, tachycardia, and muscle rigidity (Halliday, 2003; H. Rosenberg et al., 2015). While the mechanisms underlying the skeletal muscle manifestations of MH are understood in reasonable detail, there are few reports of the effects of MH mutations on neuronal function. In the absence of hyperthermia and muscle rigidity, depression of CNS electrical activity has been reported in R163C-RYR1 MH-susceptible mice after exposure to the volatile anesthetic halothane (Aleman et al., 2020). However, this has not been investigated at the cellular level, in other MH models including the *RYR1* T4826I mutation, or with modern anesthetic ethers such as isoflurane.

Volatile anesthetics modulate neurotransmission and communication between neuronal networks (Hemmings et al., 2005, 2019; Franks, 2006), including depression of synaptic transmission through both presynaptic and postsynaptic mechanisms (Hemmings et al., 2005). Volatile anesthetics inhibit activity-dependent Ca^2+^ influx into presynaptic terminals and Ca^2+^-dependent synaptic vesicle (SV) exocytosis by reducing neuronal excitability and Ca^2+^ entry. However, potential sites of action upstream of reduced Ca^2+^ entry and SV exocytosis are not fully understood.

Presynaptic endoplasmic reticulum (ER) Ca^2+^ concentration modulates Ca^2+^ entry involving ER Ca^2+^ sensing proteins. For example, reduced ER Ca^2+^ concentration is linked to reduced presynaptic Ca^2+^ influx (de Juan-Sanz et al., 2017). Ryanodine receptors (RyRs), the principal ER Ca^2+^ efflux channels, are essential for ER Ca^2+^ regulation and provide plausible targets for anesthetic action. For example, mutations in RyR1 increase Ca^2+^ efflux from skeletal muscle sarcoplasmic reticulum (SR) in response to MH-triggering agents including volatile anesthetics to initiate the pathologic features of MH including muscle rigidity and hypermetabolism. We used mice with the well characterized T4826I-RYR1 MH-susceptibility mutation (Yuen et al., 2012) to investigate the role of ER Ca^2+^ regulation in the presynaptic mechanisms of volatile anesthetic action and MH pathogenesis.

## Materials and Methods

### Animals

All animal procedures were performed in accordance with the Weill Cornell Medical College Institutional Animal Care and Use Committee regulations and conform to National Institutes of Health (Bethesda, MD) Guidelines for the Care and Use of Animals as well as ARRIVE guidelines, where appropriate. We used both wild-type (WT) Sprague Dawley rats (Charles River Strain code 400; Charles River Laboratories) and wild-type BALB/c mice (Charles River Strain code 028; Charles River Laboratories). Mice homozygous for the MH mutation T4826I-RYR1 in a BALB/c background were purchased from the University of California, Davis (Davis, CA; stock #042036-UCD) and bred for use. Homozygous T4826I-RYR1 mice were used, since 100% of homozygous T4826I-RYR1 mice develop fulminant MH. In contrast, only 17% of male heterozygous T4826I-RYR1 mice develop fulminant MH (Yuen et al., 2012).

### Primary neuron culture

Bilateral hippocampi were dissected from postnatal rats or mice (P0–P1; 0–1 d old, both sexes) and plated on poly-ornithine-coated coverslips. Neurons were maintained in culture medium containing of MEM (Thermofisher Scientific, S1200038), 30 mm glucose, 0.1 g/l bovine transferrin (Millipore, 616420), 0.25 g/l insulin, 0.3 g/l glutamine, 5–10% fetal bovine serum (Atlanta Biologicals, S11510), 2% B-27 (Thermofisher Scientific, 17504-044). Cultures were incubated at 37°C with 95% air/5% CO_2_ in a humidified incubator before imaging. Transfection was performed on day 6 or 7 *in vitro* (DIV6, DIV7) using Ca^2+^ phosphate-mediated gene transfer to transfect a low percentage of cells. Live-cell imaging was performed on DIV14–DIV19. For each experiment, neurons were derived from at least three separate culture preparations to minimize artifacts from small variations in culture conditions. The *N* in each figure corresponds to the number of cells recorded per treatment group.

### Plasmids

ER-GCaMP6-150 (Addgene, plasmid #86918), VAMP-mCherry, and synaptophysin-GCaMP6f (syn-GCaMP6) were gifts from Timothy Ryan (Weill Cornell Medicine). Synaptophysin-pHluorin (syn-pH) was a gift from Stephen Heinemann and Yongling Zhu (Salk Institute; pcDNA3-SypHluorin 2×, Addgene plasmid #37004).

### Live-cell imaging

Experiments were performed using a Zeiss Axio Observer Z1 widefield fluorescence microscope with filter cubes and LEDs for eGFP and RFP illumination (Zeiss) and an Andor iXon1 EMCCD camera sampling at 10 Hz. Coverslips with attached cells were mounted in a custom closed-bath field stimulation perfusion chamber (total volume 263 μl), with solutions and chamber maintained at 37.0 ± 0.2°C by an in-line solution heater and imaging chamber heater (Warner Instruments). Perfusion was at 1 ml/min using a custom system with a multibarrel closed syringe manifold (Warner Instruments). Only one imaging experiment was acquired per coverslip.

Standard perfusion buffer for syn-GCaMP6 and ER-GCaMP6-160 imaging was Tyrode’s solution (119 mM NaCl, 2.5 mM KCl, 1.2 mM CaCl_2_, 2.8 mM MgCl_2_, 25 mM HEPES buffered to pH 7.4, 30 mM glucose). The standard buffer for syn-pH imaging was Tyrode’s solution with 2 mM CaCl_2_ and 2 mM MgCl_2_. In experiments where external Ca^2+^ was decreased to study effects of reduced Ca^2+^, the standard buffer for imaging was Tyrode’s solution with 1 mM CaCl_2_ and 3 mM MgCl_2_. All perfusate solutions contained 10 μm 6-cyano-7-nitroquinoxaline-2,3-dione (CNQX) and 50 μm D,L-2-amino-5-phosphonovaleric acid (AP5; Tocris) to block recurrent excitation because of glutamatergic excitation. Neurons expressing syn-pH and syn-GCaMP6f were identified by their resting green fluorescence. ER-GCaMP6-150-expressing neurons were co-transfected with the presynaptic marker VAMP-mCherry to identify presynaptic boutons for selective quantification of presynaptic changes in fluorescence.

Cells transfected with syn-GCaMP6 and ER-GCaMP6-150 were electrically stimulated at 20 Hz for 1 s to mimic action potential (AP) trains of 20 AP. Cells transfected with syn-pH were stimulated with AP trains of 100 AP at 10 Hz for 10 s. Electrical stimulation was generated by field stimulation with a pulse generator (Master-9, A.M.P.I.), stimulus isolator (Model A385, World Precision Instruments) and platinum/iridium bath electrodes built into the imaging chamber to produce an electrical field of 10 V cm^−1^.

### Anesthetic solutions

Volatile anesthetic solutions were prepared daily from saturated stock solutions in Tyrode’s buffer. A 12 mM saturated stock isoflurane solution was diluted into an experimental solution equivalent to the clinically relevant dose of ∼1 minimum alveolar concentration (MAC; 0.32 mM; Taheri et al., 1991). A 6 mM saturated stock sevoflurane solution was diluted into an experimental solution equivalent to ∼1 MAC (0.48 mM). Both anesthetic solutions were perfused using gas-tight glass syringes and tubing into the imaging chamber for 5 min before imaging to allow equilibration. Perfusate samples were taken from the chamber for determination of delivered anesthetic concentrations by gas chromatography (Shimadzu GC-2010 Plus) with external standard calibration (Ratnakumari and Hemmings, 1998). The reported mean values of 0.32 mM (range 0.21–0.56 mM) isoflurane and 0.46 mM (range 0.35–0.51 mM) sevoflurane reflect mean measurements from the bath samples collected. Propofol was diluted from a 50 mM stock solution in dimethylsulfoxide (DMSO) into Tyrode’s buffer to a final concentration of 1 μm (0.002% DMSO).

### Data analysis and statistics

Live-cell imaging recordings were analyzed using ImageJ (https://imagej.nih.gov/ij/) with the TimeSeries Analyzer plugin (rsb.info.nih.gov/ij/plugins/time-series.html) to measure fluorescence over time using the Background Correction plugin (https://imagej.nih.gov/ij/plugins/download/Background_Correction_.java) to compensate for between experiment variations in background fluorescence. Transfected boutons (∼20–50 boutons per neuron) were selected from control images before drug application and analyzed using 2-μm diameter regions of interest (ROIs). ROIs were selected based on their response to control stimulation in Tyrode’s buffer. Background corrected fluorescence changes were normalized to baseline fluorescence as ΔF/F_0_ = (F – F_0_)/F_0_. Baseline fluorescence (F_0_) was defined as the mean of 10 frames before stimulus onset, and peak fluorescence (F) was defined as the mean of the five consecutive frames with the highest values immediately following electrical stimulation. Boutons with a signal-to-noise ratio more than or equal to four were used in the analysis.

Statistical significance was tested by paired or unpaired Student’s *t* tests and by paired one-way or two-way ANOVA with Tukey’s *post hoc* test, with *p* < 0.05 considered statistically significant (Table 1). Datasets were assayed for normality with the Shapiro–Wilk test. Sample size was determined by a power analysis of 0.8 with a 5% error, yielding an effect size of *n* = 6. Statistical analysis and graph preparation used GraphPad Prism v9.3 (GraphPad) and Adobe Illustrator.

**Table 1:** Statistical table

Data structure	Type of test	Confidence intervals	Figure
Normally distributed	Unpaired *t* test	−0.1384–0.2561	Figure 1
Normally distributed	Unpaired *t* test	−0.1416–0.1642	Figure 1
Normally distributed	Unpaired *t* test	−0.6070 to −0.1847	Figure 2
Normally distributed	Unpaired *t* test	−0.7229 to −0.05928	Figure 2
Normally distributed	Unpaired *t* test	−0.0864 to −0.0140	Figure 3
Normally distributed	One-way ANOVA Tukey’s *post hoc*	0.153–0.779	Figure 4
Normally distributed	One-way ANOVA Tukey’s *post hoc*	−0.0289–0.397	Figure 4
Normally distributed	One-way ANOVA Tukey’s *post hoc*	−0.448 to −0.117	Figure 4
Normally distributed	One-way ANOVA Tukey’s *post hoc*	33.8–66.3	Figure 5
Normally distributed	One-way ANOVA Tukey’s *post hoc*	23.8–45.7	Figure 5
Normally distributed	One-way ANOVA Tukey’s *post hoc*	−29.9 to −0.634	Figure 5
Normally distributed	Unpaired *t* test	−0.0961–0.229	Figure 5
Normally distributed	Unpaired *t* test	−0.203–0.258	Figure 6
Normally distributed	Unpaired *t* test	0.0134–0.393	Figure 6
Normally distributed	Two-way ANOVA Tukey’s *post hoc*	0.189–0.701	Figure 7
Normally distributed	Two-way ANOVA Tukey’s *post hoc*	0.0243–0.666	Figure 7
Normally distributed	Two-way ANOVA Tukey’s *post hoc*	−0.176–0.337	Figure 7

## Results

### Isoflurane reduces presynaptic Ca^2+^ concentration and synaptic vesicle exocytosis in wild-type and malignant hyperthermia mutant hippocampal neurons

To examine the neuronal consequences of a known human malignant hyperthermia (MH) mutation, we used homozygous knock-in mice with the homologous T4826I-RYR1 mutation (Yuen et al., 2012). This mutation increases Ca^2+^ efflux through ryanodine receptor 1 (RyR1), an endoplasmic reticulum (ER) Ca^2+^ efflux channel. Similar to human carriers, mice with the T4826I-RYR1 mutation are phenotypically normal unless challenged with an MH-triggering agent, which can produce skeletal muscle SR Ca^2+^ release and fulminant MH (Yuen et al., 2012). We examined the effects of this MH-susceptibility mutation on presynaptic function in the presence of isoflurane, by measuring presynaptic Ca^2+^ concentrations and SV exocytosis (Yuen et al., 2012).

**Figure 1.**
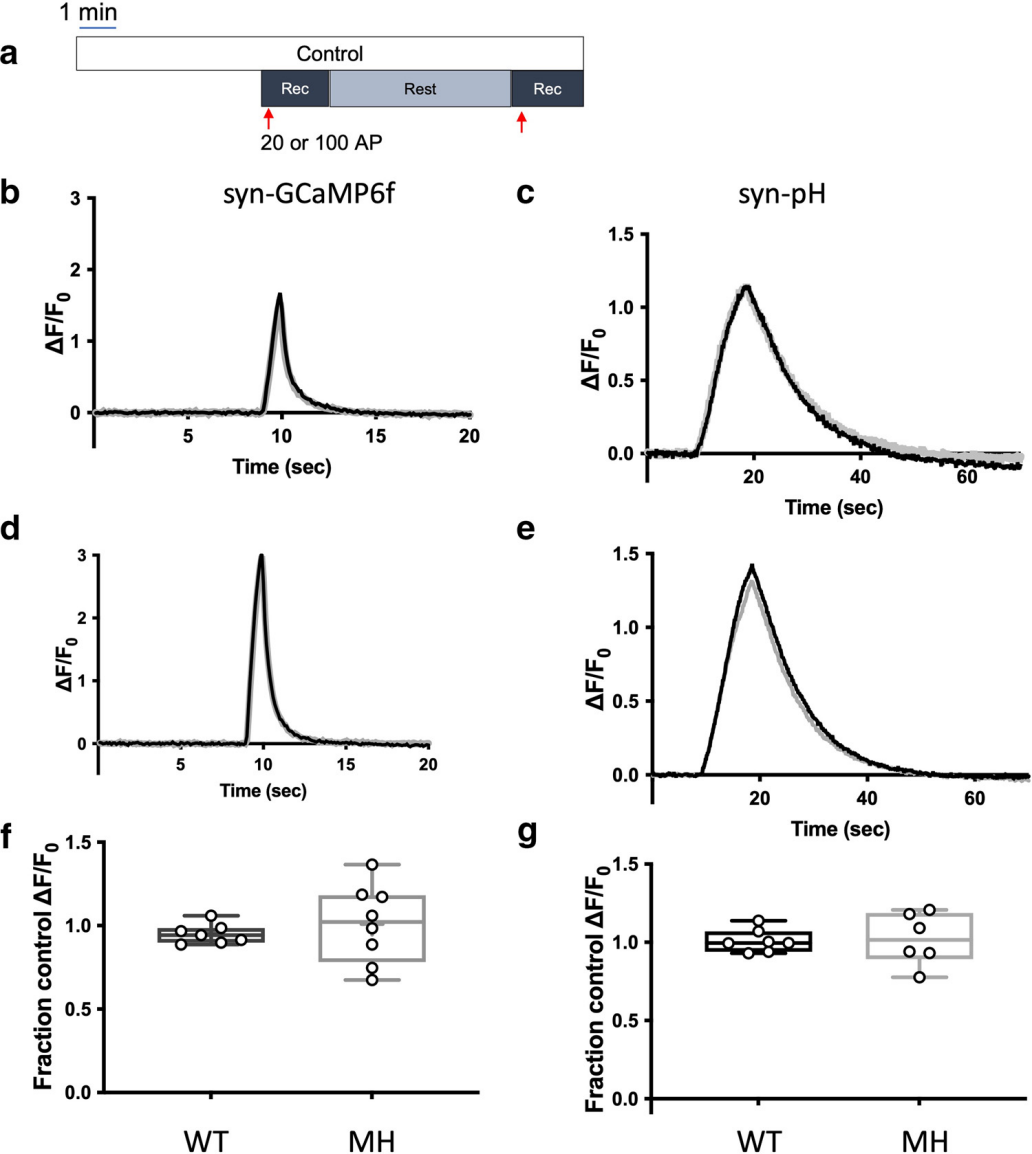
The T4826I-RYR1 malignant hyperthermia mutation does not affect presynaptic cytosolic Ca^2+^ influx or synaptic vesicle exocytosis compared with wild-type mouse hippocampal neurons. ***a***, Schematic diagram of the imaging protocol. Neurons were stimulated electrically with 20 action potentials (APs) at 20 Hz (for Ca^2+^ measurements using syn-GCaMP6f) or 100 APs at 10 Hz [for synaptic vesicle (SV) exocytosis measurements using syn-pH]. Representative average traces of (***b***) syn-GCaMP6f and (***c***) syn-pH responses to electrical stimulation in a wild-type (WT) mouse neuron in control (black) and sham (gray) conditions (50 boutons, DIV 16). Representative average traces of (***d***) syn-GCaMP6f and (***e***) syn-pH responses to electrical stimulation in a T4826I-RYR1 malignant hyperthermia susceptible mouse (MH) neuron in control (black) and sham (gray) conditions (50 boutons, DIV 16). Effects of time control stimulation on peak (***f***) syn-GCaMP6f and (***g***) syn-pH measurements in T4826I-RYR1 compared with wild-type mouse neurons normalized to their respective controls (***d***: *p* = 0.5305, unpaired *t* test, *n* = 7 WT, 8 MH; ***e***: *p* = 0.8734, unpaired *t* test, *n* = 7 WT, 6 MH).

Primary postnatal (DIV6–DIV7) mouse hippocampal neuron cultures were transfected with either syn-GCaMP6f or syn-pH for live-cell imaging. First, we measured presynaptic cytosolic Ca^2+^ (syn-GCaMP6f) and synaptic vesicle exocytosis (syn-pH) in both wild-type and T4826I-RYR1 mutant mouse neurons (Fig. 1). In control solutions, traces remain stable over two stimulations for both sensors in both wild-type and T4826I-RYR1 mutant mouse neurons (Fig. 1*b–g*).

**Figure 2.**
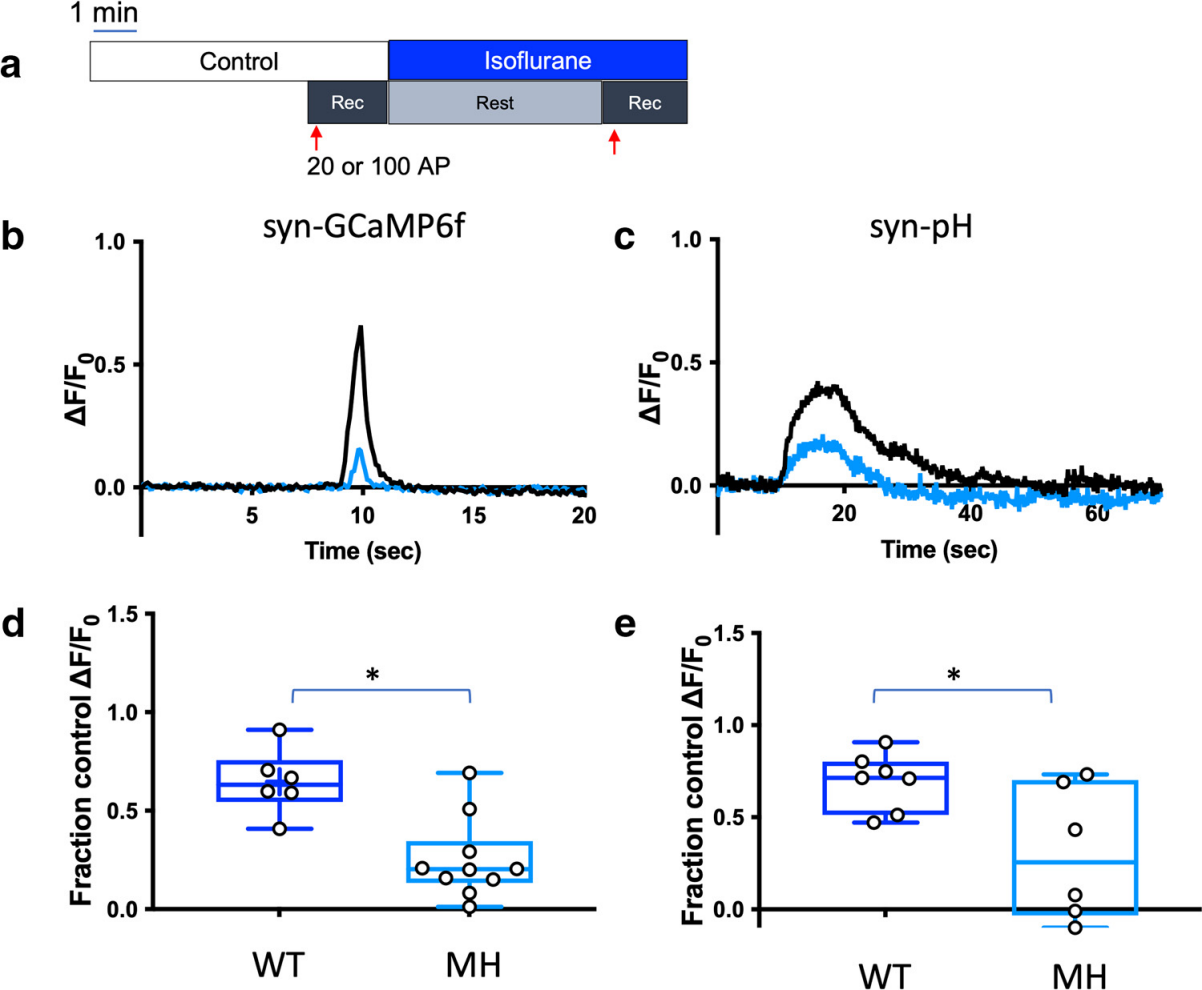
Isoflurane effects on presynaptic cytosolic Ca^2+^ influx and synaptic vesicle exocytosis are reduced in T4826I-RYR1 compared with wild-type mouse hippocampal neurons. ***a***, Schematic diagram of the imaging protocol. Neurons were stimulated electrically with 20 action potentials (APs) at 20 Hz (for Ca^2+^ measurements using syn-GCaMP6f) or 100 APs at 10 Hz [for synaptic vesicle (SV) exocytosis measurements using syn-pH]. Representative average traces of (***b***) syn-GCaMP6f and (***c***) syn-pH responses to electrical stimulation in a T4826I-RYR1 neuron in control (black) and isoflurane (blue) conditions (50 boutons, DIV 16). Effects of isoflurane on peak (***d***) syn-GCaMP6f and (***e***) syn-pH measurements in T4826I-RYR1 malignant hyperthermia susceptible (MH) compared with wild-type (WT) mouse neurons normalized to their respective controls (***d***: *p* = 0.0013, unpaired *t* test, *n* = 6 WT, 10 MH; ***e***: *p* = 0.0250, unpaired *t* test, *n* = 7 WT, 6 MH).

Figure 2*a* shows the protocol used to image neurons transfected with syn-GCaMP6f or syn-pH and treated with a clinically relevant concentration of isoflurane. In wild-type mouse neurons, isoflurane depressed stimulation-evoked increases in presynaptic cytosolic Ca^2+^ and synaptic vesicle exocytosis (Fig. 1*b*,*c*) to a degree comparable to that described in rat neurons (Baumgart et al., 2015). The T4826I-RYR1 mutation markedly enhanced isoflurane inhibition of both depolarization evoked increases in presynaptic cytosolic Ca^2+^ concentration (Fig. 2*b*) and SV exocytosis (Fig. 2*c*) compared with wild-type neurons, with greater inhibition by isoflurane of presynaptic cytosolic Ca^2+^ (*p* = 0.0015; Fig. 2*d*) and exocytosis (*p* = 0.0148; Fig. 2*e*) in mutant neurons. Wild-type mouse neurons exhibited 35% depression of stimulation-evoked increases in presynaptic cytosolic Ca^2+^ concentration and 41% depression of SV exocytosis (Fig. 2*d*,*e*), while T4826I-RYR1 neurons treated with isoflurane exhibited 75% depression of presynaptic cytosolic Ca^2+^ concentration (WT isoflurane vs MH isoflurane: *p* = 0.0013; Fig. 2*d*) and 63% depression of SV exocytosis (WT isoflurane vs MH isoflurane: *p* = 0.0250; Fig. 2*e*). These results suggest that the T4826I-RYR1 MH mutation enhance the actions of isoflurane and contributes to neuronal dysfunction in MH-susceptible neurons in response to isoflurane through alterations in presynaptic ER Ca^2+^ regulation. These results also indicate that RyR1 makes a functionally important contribution to synaptic function in hippocampal neurons.

### Isoflurane reduces endoplasmic reticulum Ca^2+^ concentration independent of inhibition of Ca^2+^ influx

**Figure 3.**
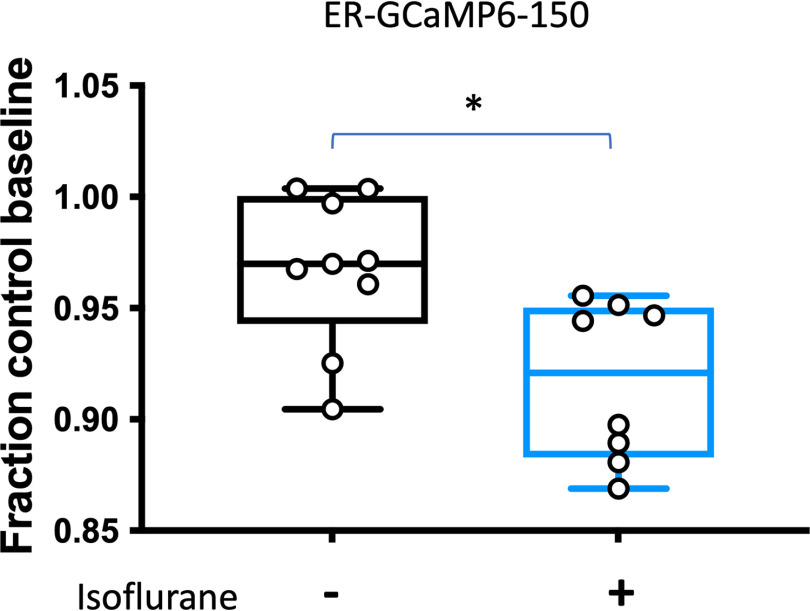
Isoflurane reduces resting endoplasmic reticulum Ca^2+^ concentration in rat hippocampal neurons. Baseline ER-GCaMP6-150 fluorescence was measured in wild-type rat neurons treated with isoflurane compared with a separate time control neuron [*p* = 0.0098, unpaired *t* test, *n* = 9 (left), *n* = 7 (right)].

**Figure 4.**
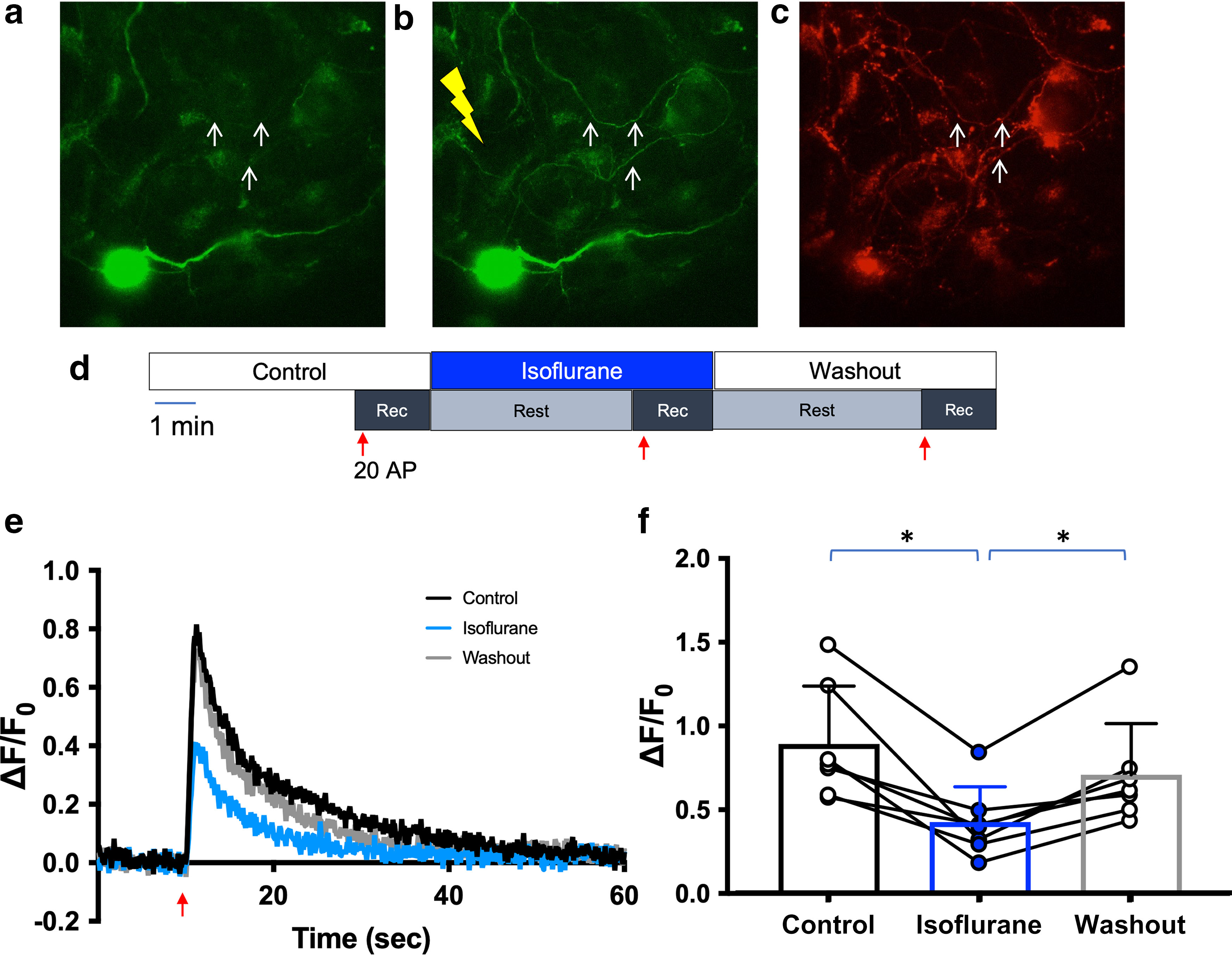
Isoflurane reduces stimulation-evoked increases in endoplasmic reticulum Ca^2+^ concentration. ***a–c***, Fluorescence images of a rat hippocampal neuron co-transfected with ER-GCaMP6-150 (green) and VAMP mCherry (red). Live-cell imaging measuring [Ca^2+^]ER (***a***) before and (***b***) during electrical stimulation. ***c***, Snapshot of the presynaptic marker VAMP-mCherry. ***d***, Schematic diagram of the protocol used in ***b***, ***c***. ***e***, Representative average traces of ER-GCaMP6-150 fluorescence changes with 20 action potentials (APs) at 20 Hz stimulations for control, isoflurane, and washout conditions (*n* = 1, 50 boutons, DIV 16). ***f***, Peaks of ER-GCaMP6-150 fluorescence over three stimulations of 20 AP each at 20 Hz for control (white circle) and isoflurane (blue circle) conditions. Control versus isoflurane, *p* = 0.0091; isoflurane versus washout, *p* = 0.0047; control versus washout *p* = 0.9967; one-way ANOVA, *n* = 8.

These findings led us to investigate the role of ER Ca^2+^ as a target for the presynaptic effects of isoflurane, using rat neuron cultures to optimize the protocols. Using ER-GCaMP6-150, a genetically encoded, ER-targeted Ca^2+^ sensor, we found that isoflurane significantly depressed baseline ER Ca^2+^ concentration in wild-type rat hippocampal neurons by 5% (*p* = 0.0098; Fig. 3). To restrict analysis to presynaptic boutons, we used co-transfection with the presynaptic marker VAMP-mCherry to analyze boutons co-expressing ER-GCaMP6-150 and VAMP-mCherry (Fig. 4*a–c*). Using the protocol shown in Figure 4*d*, isoflurane reversibly inhibited stimulation-evoked increases in ER Ca^2+^ concentration by ∼57% (*p* = 0.0091; Fig. 4*e*,*f*).

**Figure 5.**
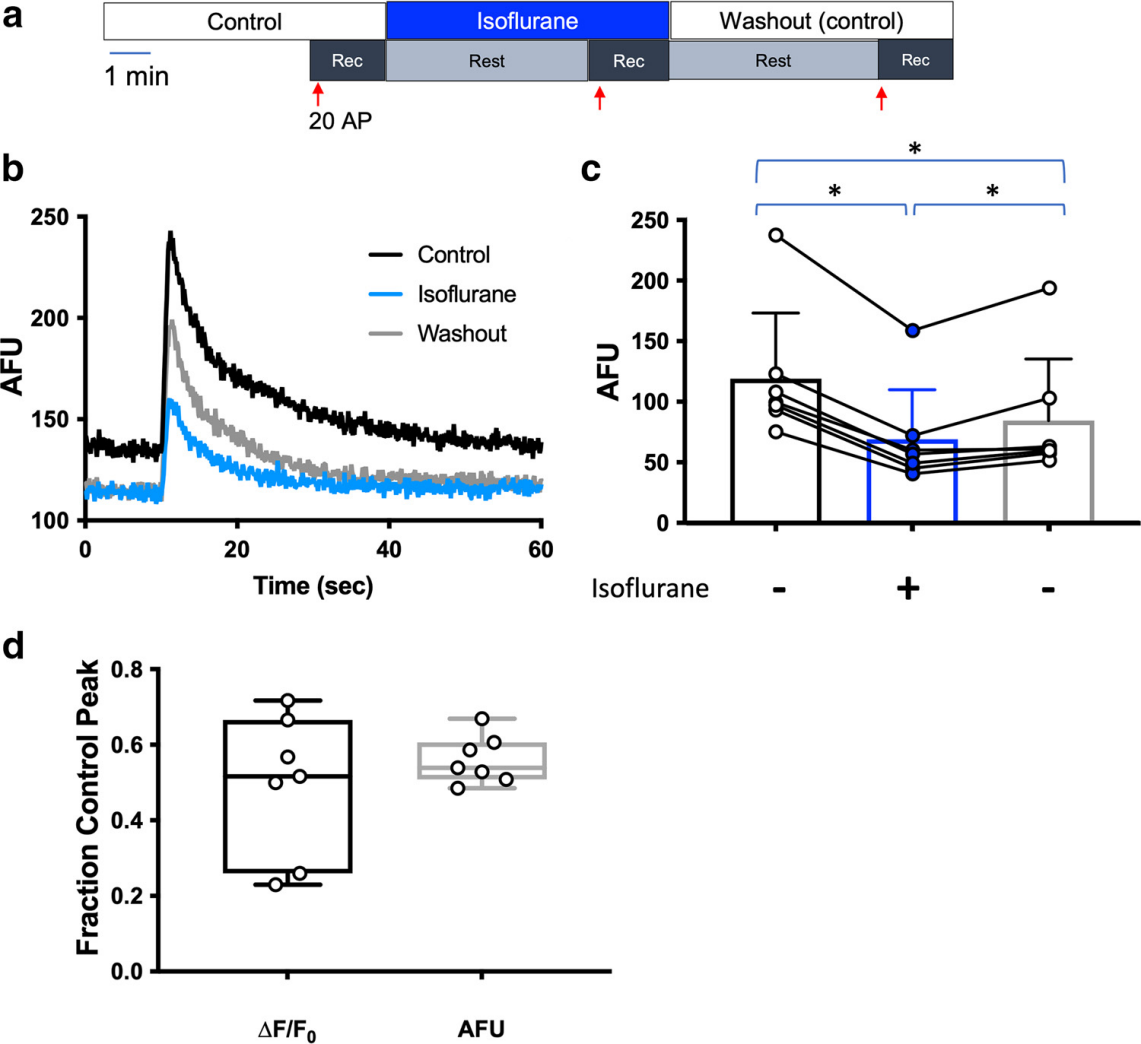
Isoflurane depression of baseline ER Ca^2+^ concentration was not sufficient to disrupt normalization using ΔF/F0. ***a***, Schematic diagram of the protocol used in ***b***, ***c***. ***b***, Representative average traces in arbitrary fluorescence units (AFU) of ER-GCaMP6-150 fluorescence changes with 20 action potentials (APs) at 20 Hz stimulations for control, isoflurane, and washout conditions (*n* = 1, 50 boutons, DIV 16). ***c***, Arbitrary fluorescence unit (AFU) peaks of ER-GCaMP6-150 fluorescence over three stimulations of 20 AP each at 20 Hz for control (white circle) and isoflurane (blue circle) conditions. (control vs isoflurane *p* = 0.0002, isoflurane vs washout *p* = 0.0002, control vs washout *p* = 0.0425, one-way ANOVA, *n* = 8). ***d***, Box and whisker plot comparing the same data set measured two ways: ΔF/F0 or total AFU of isoflurane-treated normalized to its respective control condition (*p* = 0.3457, paired *t* test, *n* = 8).

Since isoflurane reduced both baseline and stimulation-evoked increases in ER Ca^2+^ concentration, we reanalyzed data from Figure 4*b*,*c* using arbitrary fluorescence units (AFUs) rather than normalized F to mitigate a potential effect of the isoflurane-induced change in baseline fluorescence on ΔF/F_0_. The degree of inhibition of unnormalized F was comparable to that of normalized ΔF/F_0_ (*p* = 0.0002; Fig. 5*b*,*c*). There was no significant difference between results obtained using ΔF/F_0_ values normalized to control values or raw AFU values normalized to control values in the same set of isoflurane-treated neurons (Fig. 5*d*). We therefore used ΔF/F_0_ for subsequent experiments.

**Figure 6.**
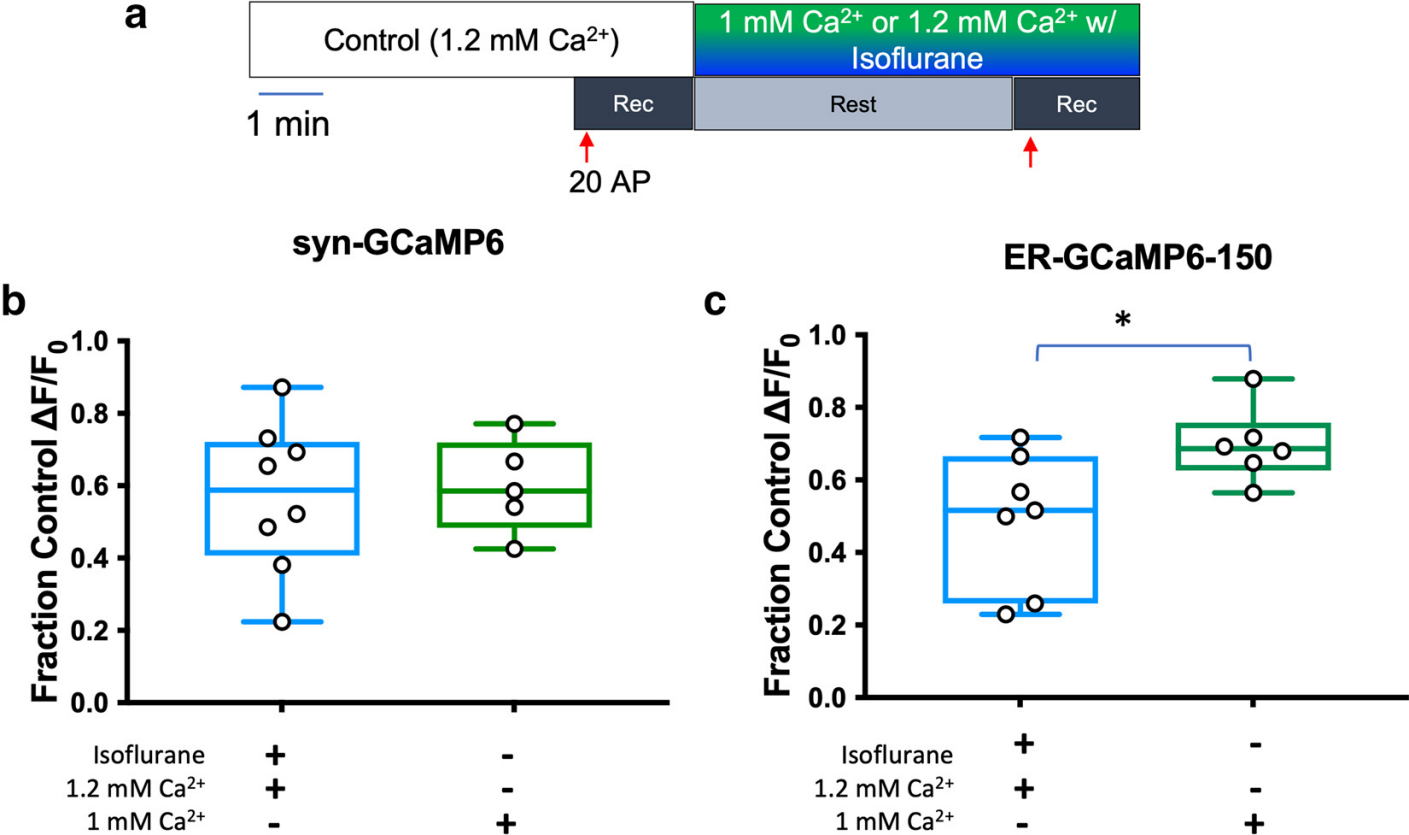
Reduction of stimulation-evoked increase in endoplasmic reticulum Ca^2+^ concentration by isoflurane is not dependent on reduced Ca^2+^ influx. ***a***, Schematic diagram of the protocol used. Box and whisker plot comparing (***b***) syn-GCaMP6f or (***c***) ER-GCaMP6-150-transfected cells stimulated with 20 action potentials (APs) at 20 Hz with 0.30 (±0.11) mM isoflurane normalized to control with 1.2 mM Ca^2+^ Tyrode’s solution, or control with 1 mM Ca^2+^ Tyrode’s solution normalized to 1.2 mM Ca^2+^ Tyrode’s solution [***b***: *p* = 0.7963, unpaired *t* test, *n* = 8 (left), 5 (right); ***c***: *p* = 0.0380, unpaired *t* test, *n* = 7 (left), 6 (right)].

We hypothesized that depression of presynaptic Ca^2+^ influx by isoflurane triggers a reduction in the amount of cytosolic Ca^2+^ available for sequestration by the ER, thereby reducing ER Ca^2+^ uptake and reducing the increase in intraluminal ER Ca^2+^ concentration in response to electrical stimulation. To test this mechanism, we lowered extracellular Ca^2+^ from 1.2 mM to 1 mM to reduce the stimulation-evoked increase in presynaptic cytosolic Ca^2+^ to a similar degree as observed with isoflurane exposure. This reduction in extracellular Ca^2+^ concentration led to a 40% reduction in presynaptic Ca^2+^ influx in wild-type rat hippocampal neurons in the absence of isoflurane, which is comparable to the reduction produced by 0.30 mM isoflurane (Fig. 6*b*). However, compared with the reduction in ER Ca^2+^ observed with reduced extracellular Ca^2+^, the degree of isoflurane inhibition of the stimulation-evoked increase in ER Ca^2+^ concentration was greater (*p* = 0.0380; Fig. 6*c*). Thus, the reduction in evoked ER Ca^2+^ concentration by isoflurane is not fully attributable to its inhibition of presynaptic cytosolic Ca^2+^ influx, supporting an additional mechanism(s) of action.

### The T4826I-RYR1 malignant hyperthermia mutation potentiates volatile anesthetic depression of stimulation-evoked increases in endoplasmic reticulum Ca^2+^

**Figure 7.**
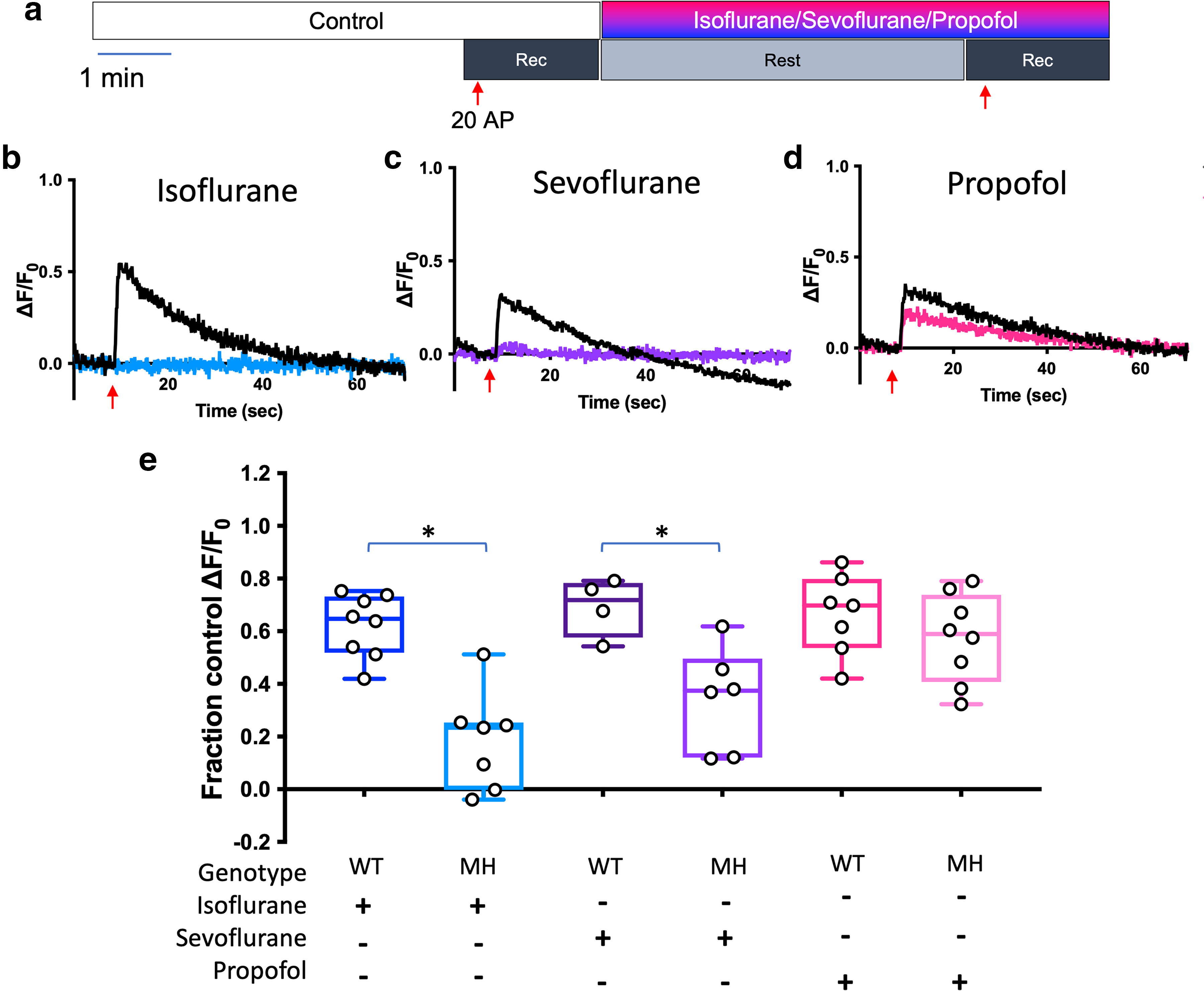
Volatile anesthetics potentiate stimulation-evoked reduction of endoplasmic reticulum Ca^2+^ concentration in T4826I-RYR1 mouse neurons. ***a***, Schematic diagram of the protocol. Neurons were stimulated electrically with 20 action potentials (APs) at 20 Hz. Representative average traces of T4826I-RYR1 malignant hyperthermia (MH) mouse neurons transfected with ER-GCaMP6-150 perfused with (***b***) 0.34 mM isoflurane, (***c***) 0.46 mM sevoflurane, or (***d***) 1 μm propofol. Peak ER-GCaMP6-150 effect of isoflurane, sevoflurane, or propofol on T4826I-RYR1 compared with wild-type (WT) mouse neurons normalized to their respective controls (***e***: *p* = WT isoflurane vs MH isoflurane: 0.0002, WT sevoflurane vs MH sevoflurane: 0.0295, WT propofol vs MH propofol: 0.9258, two-way ANOVA, *n* = 8, 7, 4, 6, 7, 8, respectively).

We compared the effects of isoflurane on stimulation-evoked increases in ER Ca^2+^ in wild-type and T4826I-RYR1 mutant mouse hippocampal neurons to explore the impact of this MH-susceptibility mutation on anesthetic effects. Isoflurane produced a large reduction in the stimulation-evoked increase in ER Ca^2+^ concentration in T4826I-RYR1 mouse hippocampal neurons compared with wild-type neurons (*p* = 0.0002; Fig. 7*b*,*e*). Sevoflurane, another volatile anesthetic trigger of MH, had a similar effect (*p* = 0.0295; Fig. 7*c*,*e*). Both volatile anesthetics reduced stimulation evoked increases in ER Ca^2+^ concentration to a greater extent in T4826I-RYR1 neurons (MH isoflurane vs MH control 80% inhibition: *p* < 0.0001; MH sevoflurane vs MH control 63% inhibition: *p* < 0.0001) compared with in wild-type neurons (WT isoflurane vs WT control 31% inhibition: *p* = 0.0009; WT sevoflurane vs WT control 23% inhibition: *p* = 0.0367). In contrast, propofol, an intravenous anesthetic that is not a trigger of MH, reduced evoked ER Ca^2+^ concentration in wild-type neurons but did not potentiate the reduction in T4826I-RYR1 mutant neurons (Fig. 7*d*,*e*; not significant; WT propofol vs WT control 27% inhibition: *p* = 0.0052; MH propofol vs MH control 39% inhibition: *p* = 0.0011). The effects of isoflurane or sevoflurane on evoked increases in ER Ca^2+^ in T4826I-RYR1 neurons were significant compared with their effects on ER Ca^2+^ in wild-type neurons (Fig. 7*e*), while the effect of propofol on ER Ca^2+^ was not different between T4826I-RYR1 and wild-type neurons (*p* = 0.9258;Fig. 7*e*). These results suggest that this MH-susceptibility mutation has marked effects on presynaptic Ca^2+^ handing in the presence of triggering volatile anesthetics, but not nontriggering intravenous anesthetics.

### Viability of T4826I-RYR1 mouse neurons

**Figure 8.**
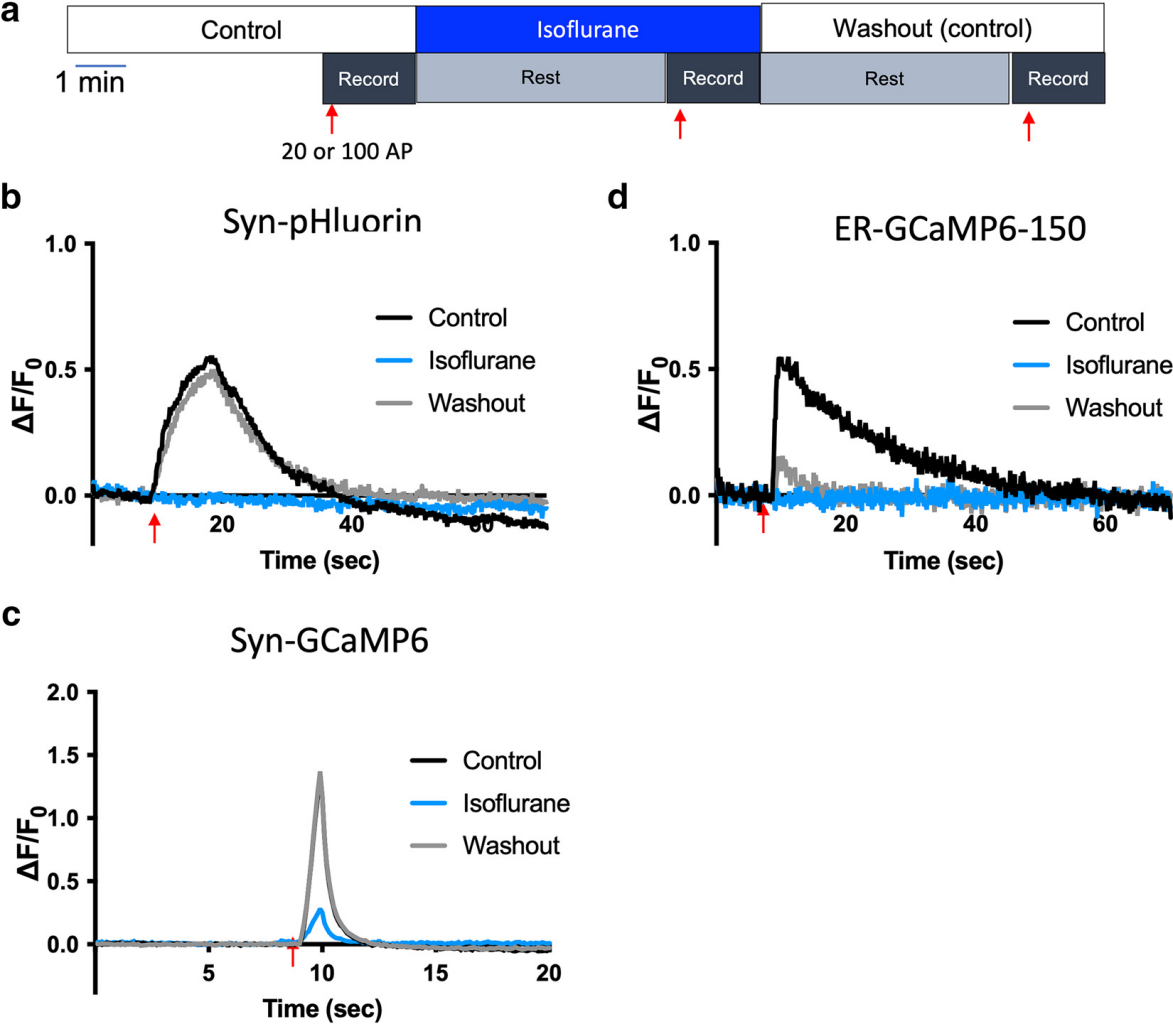
T4826I-RYR1 malignant hyperthermia susceptible mouse neurons remain stable and responsive over repeated stimulations. ***a***, Schematic diagram of the protocol. Neurons were stimulated electrically with 20 action potentials (APs) at 20 Hz. Representative average traces of T4826I-RYR1 mouse neurons perfused with control solution transfected with (***b***) syn-pH, (***c***) syn-GCaMP6f, or (***d***) ER-GCaMP6-150.

**Figure 9.**
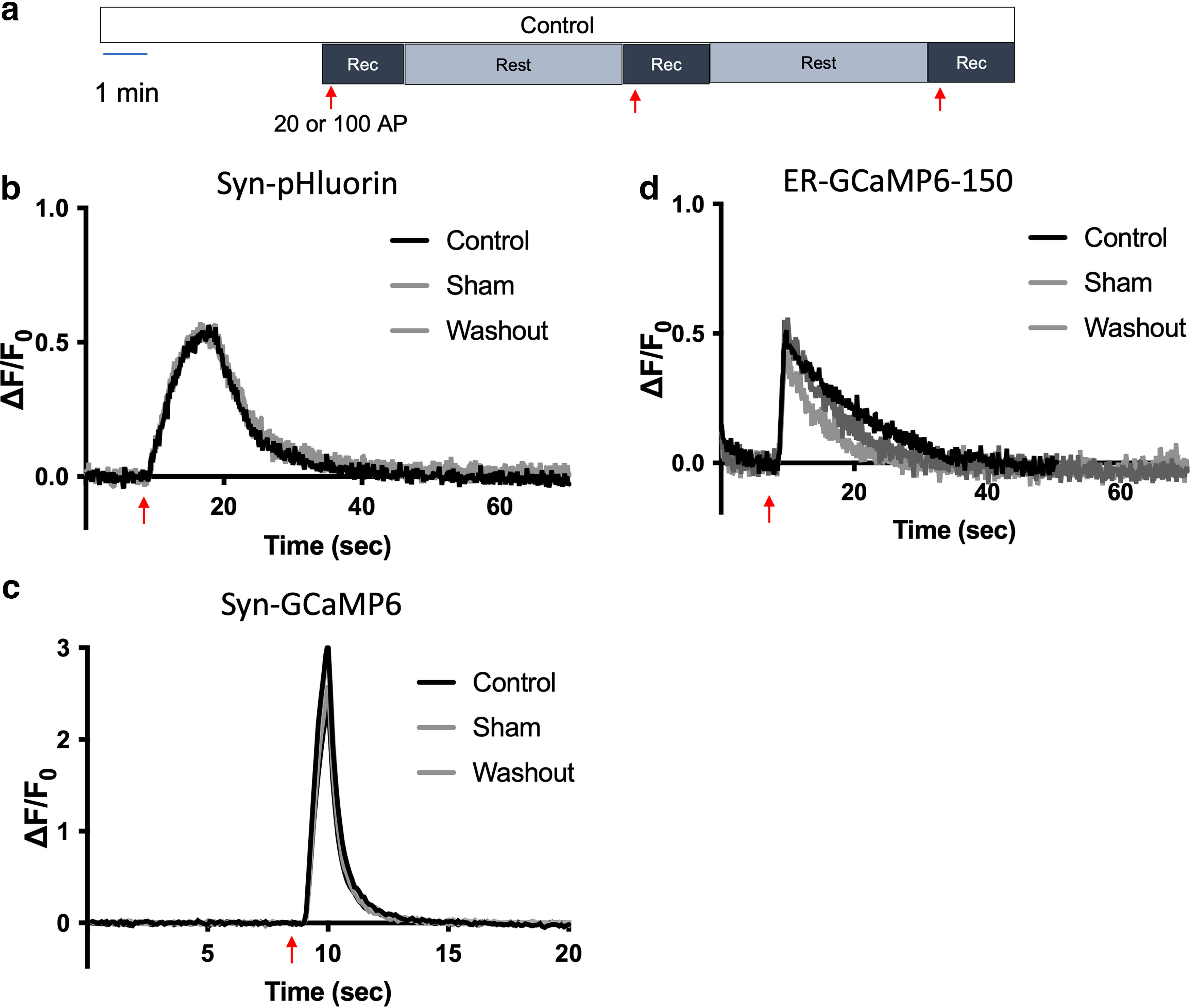
Enhanced isoflurane-induced inhibition of stimulation-evoked presynaptic synaptic vesicle exocytosis, cytosolic Ca^2+^ concentration, and ER Ca^2+^ concentration in T4826I-RYR1 malignant hyperthermia susceptible mouse neurons is reversible. ***a***, Schematic diagram of the protocol. Neurons were stimulated electrically with 20 action potentials (APs) at 20 Hz. Representative average traces of T4826I-RYR1 mouse neurons perfused with isoflurane transfected with (***b***) syn-pH, (***c***) syn-GCaMP6f, or (***d***) ER-GCaMP6-150.

Given the finding that the T4826I-RYR1 MH-susceptibility mutation greatly enhances isoflurane depression of electrical stimulation-evoked increases in presynaptic Ca^2+^, SV exocytosis, and ER Ca^2+^ concentration, it was important to show that T4826I-RYR1 neurons remain viable after isoflurane treatment, i.e., that the effects are not because of enhanced neurotoxicity. Most neurons exhibited a return in responsiveness to stimulation after washout of isoflurane (Fig. 8). There was a complete return of responsiveness for SV exocytosis (syn-pH) and presynaptic Ca^2+^ concentration (syn-GCaMP6) in T4826I-RYR1 neurons (Fig. 8*b*,*c*). However, there was only a partial return of responsiveness in ER Ca^2+^ (ER-GCaMP6-150; Fig. 8*d*). It is likely that the 5-min washout period was insufficient for recovery of ER Ca^2+^ in T4826I-RYR1 neurons. Together these findings show that isoflurane depression of stimulation-evoked increases in ER Ca^2+^ concentration, presynaptic Ca^2+^ concentration, and SV exocytosis in T4826I-RYR1 neurons is not the result of irreversible cell death. Moreover, the mutant neurons were not more sensitive to electrical stimulation than wild-type neurons, and the mutant neurons remained stable and responsive over multiple electrical stimulations in the absence of isoflurane (Fig. 9).

## Discussion

General anesthetics induce a complex drug-induced coma that has intrigued neuropharmacologists since its initial demonstration in 1846 because of its reversible effects on memory, consciousness, and movement in response to pain (Hemmings et al., 2019). General anesthetics have marked effects on synaptic transmission, but our understanding of the mechanisms involved in their presynaptic effects remain incomplete. Moreover, the role anesthetic effects on presynaptic neuronal intracellular Ca^2+^ store regulation has not been investigated previously. Here, we show that volatile anesthetics, but not propofol, disrupt neuronal ER Ca^2+^ handling, and that these effects are enhanced in a mouse model of malignant hyperthermia susceptibility. We identified inhibitory effects of isoflurane on stimulation-evoked increases in neuronal ER Ca^2+^ concentration in wild-type neurons, demonstrating presynaptic ER Ca^2+^ handing as a neuronal target for anesthetic effects. Moreover, we showed that the inhibitory effects of isoflurane on stimulation-evoked increases in presynaptic ER Ca^2+^, cytosolic Ca^2+^, and SV exocytosis are enhanced in the well characterized T4826I-RYR1 mouse model of human malignant hyperthermia.

Most previous studies of anesthetic effects on the ER have focused on its role in modulating cell viability (Q.J. Wang et al., 2008; Zhai et al., 2015; Liu et al., 2016). Our studies are the first to analyze the functional impact of anesthetic effects on ER Ca^2+^ on neuronal function in intact hippocampal neurons. Using fluorescent biosensors to measure ER Ca^2+^ in live neurons, we found that isoflurane reduced both resting baseline and electrical activity-evoked increases in presynaptic ER Ca^2+^ concentration. The mechanisms of isoflurane effects on ER Ca^2+^ regulation are better understood in other cell types, particularly in skeletal muscle and cardiomyocytes (Davies et al., 2000; Pabelick et al., 2004; Dworschak et al., 2006; Klingler et al., 2014), but have not been characterized in neurons. Anesthetic depression of stimulation-evoked ER Ca^2+^ could be attributed to reductions in presynaptic cytosolic Ca^2+^ influx, but by reducing external Ca^2+^ concentration to mimic the isoflurane-induced reduction in presynaptic cytosolic Ca^2+^ we were able to distinguish between the known inhibition of presynaptic cytosolic Ca^2+^ by isoflurane and a distinct effect to reduce ER Ca^2+^ concentration. These data indicate a distinct mechanism underlying modulation of ER Ca^2+^ by volatile anesthetics mediated by RyR1.

Possible mechanisms for the presynaptic effects of isoflurane on ER Ca^2+^ regulation include a direct effect on ER Ca^2+^ regulation that leads to depression of presynaptic cytosolic Ca^2+^ and SV exocytosis, or separate effects on ER Ca^2+^ and presynaptic cytosolic Ca^2+^ concentration regulation that combine to inhibit SV exocytosis. The first possibility suggests that isoflurane depression of increases in ER Ca^2+^ is upstream of its depression of presynaptic cytosolic Ca^2+^ influx. Pharmacological inhibition of Ca^2+^ uptake into the ER by the SERCA pump reduces stimulation-evoked increases in both presynaptic cytosolic Ca^2+^ and SV exocytosis involving a temperature-dependent positive feedback loop in which ER Ca^2+^ content controls presynaptic cytosolic Ca^2+^ influx and SV exocytosis (de Juan-Sanz et al., 2017). Although the mechanism is unclear, STIM1, an ER Ca^2+^ sensor, is essential for normal CNS function; a conditional STIM1 knock-out results in marked learning defects and impaired cerebellum-regulated motor activity (Hartmann et al., 2014; Garcia-Alvarez et al., 2015; de Juan-Sanz et al., 2017). Cognitive dysfunction and impaired motor activity are also features of isoflurane anesthesia, so depression of ER Ca^2+^ through enhanced efflux via RyRs (Glover et al., 2004) might activate the STIM1-mediated positive feedback loop contributing to depression of presynaptic cytosolic Ca^2+^ and SV vesicle exocytosis, similar to the effect of SERCA inhibition (de Juan-Sanz et al., 2017). The second possibility suggests that the isoflurane effect on ER Ca^2+^ is secondary to its effect on presynaptic Ca^2+^ concentration. These mechanisms might each contribute to isoflurane depression of SV exocytosis, ER Ca^2+^ concentration through the STIM1-mediated positive feedback loop, and presynaptic cytosolic Ca^2+^ because of reduced Ca^2+^ entry. Distinguishing between these mechanisms and their individual contributions will be technically challenging as they are inextricably linked, but our results indicate multiple mechanisms.

Isoflurane depression of ER Ca^2+^ increases involves a distinct mechanism that could also contribute to some of its undesirable neurologic side effects (e.g., neurotoxicity, cognitive dysfunction). Hereditary ER dysfunction has been associated with cognitive dysfunction (Liu et al., 2012); if isoflurane effects on ER Ca^2+^ contribute to cognitive dysfunction, blocking or reducing its effects on ER Ca^2+^ might ameliorate this undesirable aspect of anesthesia (Rundshagen, 2014).

The mechanisms by which isoflurane reduces ER Ca^2+^ concentration could involve reduced uptake and/or enhanced efflux (Glover et al., 2004). Isoflurane could activate ER Ca^2+^ uptake or efflux pathways, including smooth endoplasmic reticulum Ca2+ ATPase (SERCA), inositol triphosphate receptor (IP_3_R), or RYR mediated mechanisms (Lytton et al., 1992). It is unlikely SERCA is the major mechanism for the effects we observed, as SERCA is not known to be modulated by isoflurane or directly affected by the T4826I-RYR1 mutation. Because of the known effects of volatile anesthetics on RyR1 in skeletal muscle in MH, direct interaction with RyR1 is likely involved in the neuronal effects of volatile anesthetics on ER Ca^2+^. A recent pilot study in heterozygous R163C-RYR1 MH-susceptible mice showed enhanced suppression of CNS electrical activity by the volatile anesthetic halothane *in vivo* consistent with CNS effects of an MH-associated RYR1 mutation (Aleman et al., 2020). Caffeine, an RyR agonist, enhanced halothane suppression of EEG power in R163C-RYR1 mice, suggesting that a volatile anesthetic has functional CNS phenotype in MH-susceptible mice. However, RyR1 might not be the sole mechanism contributing to this phenotype, as volatile anesthetics are promiscuous drugs that suppress EEG power, and it is possible that their effects involve interactions with multiple Ca^2+^ influx and efflux pathways (Baumgart et al., 2015). Isoflurane might also directly or indirectly alter production or localization of endogenous modulators of presynaptic Ca^2+^ channels and transporters.

Specific mutations that alter SR Ca^2+^ handling in skeletal muscle confer MH susceptibility (Ali et al., 2003). We hypothesized that RyRs are involved in neuronal function though presynaptic ER Ca^2+^ regulation, and that MH-susceptibility mutations also alter the neuronal effects of volatile anesthetics mediated by ER Ca^2+^ regulatory pathways. Most MH-susceptibility mutations occur in *RYR1*, which encodes an SR/ER Ca^2+^ efflux channel (H. Rosenberg et al., 2015). RyR1 is the predominant isoform expressed in skeletal muscle, and appears to be expressed in brain as well (Hakamata et al., 1992; Furuichi et al., 1994), but the effects of volatile anesthetics on neurons expressing MH-susceptibility mutations had not been investigated previously.

RyR isoform localization and function in neurons have not been clearly resolved. All three isoforms (RyR1–RyR3) are expressed in brain, however their roles in neuronal function have not been characterized. RyR expression has been detected in several hippocampal neuron compartments including in presynaptic terminals, dendritic spines, and somata (Nakanishi et al., 1992; Seymour-Laurent and Barish, 1995; Hertle and Yeckel, 2007; Shimizu et al., 2008; Wayman et al., 2012). However, because of poor antibody specificity, subcellular localization of RyR1 and other RyR isoforms in hippocampal neurons is not well undefined (Hiess et al., 2022). Our findings suggest that RyR1 is functionally expressed in hippocampal neurons, a significant advance in understanding the neuronal roles of RyRs. Contradictory results have suggested either the presence or absence of RyR1 in the hippocampus (Mori et al., 2000; Galeotti et al., 2008). We found that an MH-susceptibility mutation in RyR1 led to enhanced anesthetic-induced depression of electrical stimulation-evoked increases in presynaptic ER Ca^2+^, cytosolic Ca^2+^, and SV exocytosis. We also provide novel evidence that RyR1 plays a critical role in synaptic transmission of hippocampal neurons.

T4826I-RYR1 mutant mice are a suitable model for studying MH susceptibility: they have no overt phenotype in the absence of volatile anesthetics, as is typical in humans with this and other MH-susceptibility mutations. We used homozygous T4826I-RYR1 mice since 100% of homozygous T4826I-RYR1 mice develop fulminant MH while only 17% of male and no female heterozygous T4826I-RYR1 mice develop fulminant MH in response to halothane as a trigger (Yuen et al., 2012). Such complete penetrance is essential to interpretation of our *in vitro* findings. Our results show that the T4826I-RYR1 mutation exacerbates isoflurane depression of stimulus-evoked increases in ER Ca^2+^, as well as of presynaptic cytosolic Ca^2+^ and SV exocytosis. This effect was specific to the volatile anesthetics sevoflurane and isoflurane, while propofol, a mechanistically distinct intravenous anesthetic, had no significant effect on ER Ca^2+^ in MH-susceptible neurons compared with wild-type neurons. This is consistent with the MH triggering activity of volatile anesthetics, which can be lethal in triggering MH, while propofol is safe for MH-susceptible patients (M.B. Rosenberg, 1991; Gupta et al., 2021). The marked effects of volatile anesthetics are not the result of cell death or loss of responsivity, as the effects were largely reversible.

**Figure 10.**
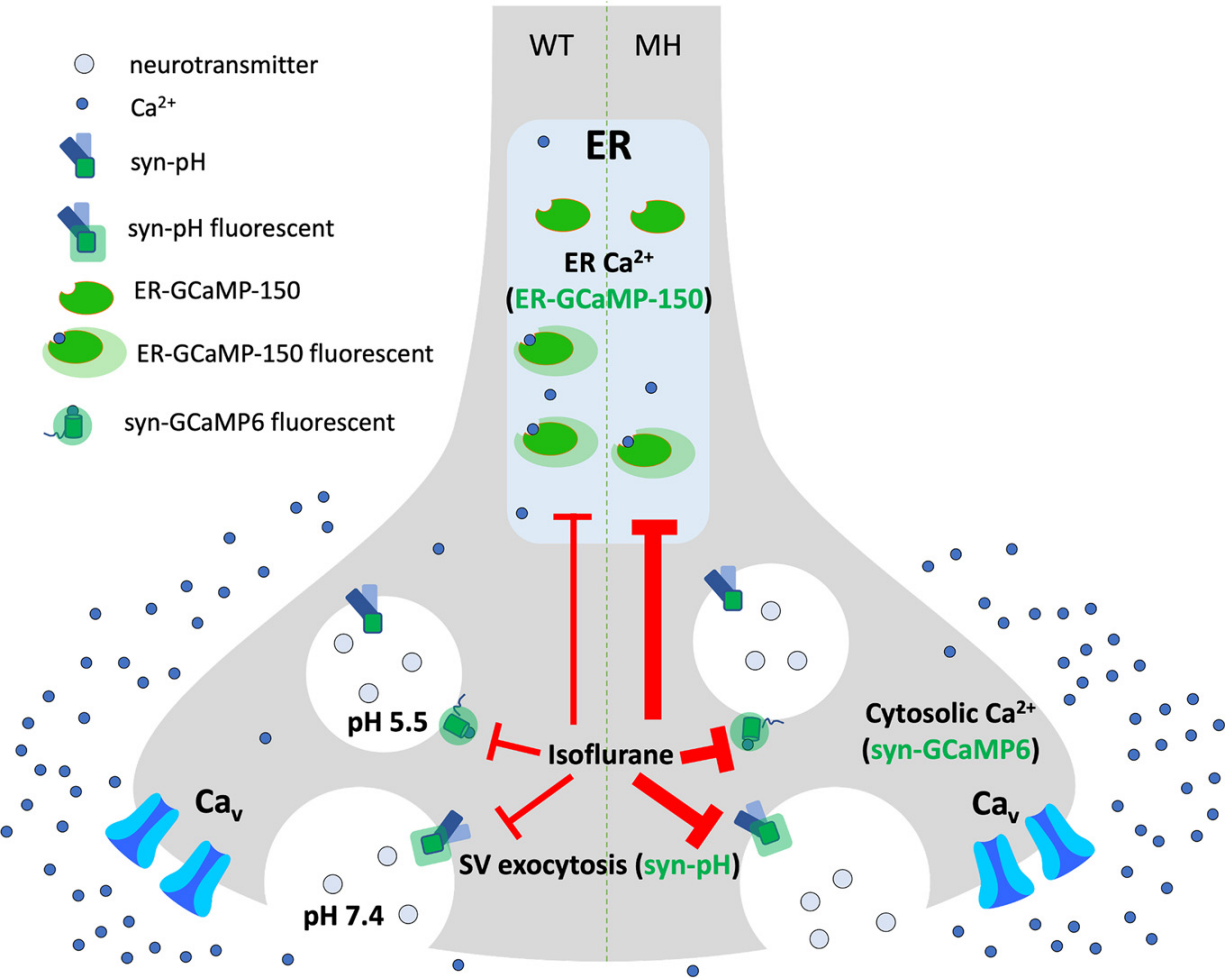
Overview of possible presynaptic volatile anesthetic mechanisms. Schematic diagram of isoflurane depression of presynaptic ER Ca^2+^ concentration, cytosolic Ca^2+^ concentration, and synaptic vesicle exocytosis in wild-type (left half) and MH-susceptible (right half) mice (ER: endoplasmic reticulum; MH: malignant hyperthermia; syn-pH: synaptophysin-pHlourin; syn-GCaMP6: synaptophysin-GCaMP6f, SV: synaptic vesicle).

Our results imply that an MH episode might involve direct neurologic effects, including depression of synaptic transmission with possible short-term and long-term effects not yet investigated clinically (Fig. 10). Heat-induced central nervous system (CNS) damage because of MH has been reported (Gore and Isaacson, 1949). However, the pattern of brain injury seen in MH differs from the pattern seen in hypoxic-ischemic brain injury (Forrest et al., 2015). This coupled with our results suggests that MH-induced CNS injury is not exclusively because of hyperthermia-induced cytotoxicity, but also involves direct effects on neuronal Ca^2+^ regulation.

Treatment of MH involves early administration of dantrolene, an RyR antagonist. Although dantrolene is lipid soluble with a molecular mass of 314, properties that generally predict blood-brain barrier permeability, it exhibits limited CNS penetration (J. Wang et al., 2020), and there is conflicting evidence for its to cross the blood-brain barrier (Meyler et al., 1981; Wuis et al., 1989). Future studies should focus on elucidating the impact of MH on neuronal function and cytotoxicity, as well as mitigating possible dysfunction and neurotoxicity in MH, and possibly other neurologic diseases.

### Limitations

Use of primary dissociated neurons as an experimental model does not fully recapitulate cellular function and interactions *in vivo*. Our focus on presynaptic function might have overlooked other relevant mechanisms observed in intact neuronal circuits, including postsynaptic and glial contributions. We focused our studies on the hippocampus as a model given the large body of data available on its fundamental neurophysiology, sensitivity to general anesthetics, and critical role in anesthetic actions (Rudolph and Antkowiak, 2004; D.S. Wang and Orser, 2011; Hemmings et al., 2019). The fundamental cellular mechanisms of Ca^2+^ regulation are shared between many neuron types, however observations in hippocampal neurons might not translate to other neuronal types because of cell type-specific differences. Use of a single RyR1 mutation model for MH susceptibility from the many known human mutations (Hopkins, 2011) is a potential limitation, however there are limited viable animal models for human MH susceptibility that are as well characterized as the T4826I-RYR1 mutation (Yuen et al., 2012).

### Future directions

Understanding the mechanisms of anesthetic effects on ER Ca^2+^ has implications for the presynaptic mechanisms of volatile anesthetics. Future studies will examine the role of the STIM1 feedback loop in anesthetic mechanisms using knock-down models (de Juan-Sanz et al., 2017). It is also important to understand the effects of dantrolene on anesthetic effects in MH-susceptible neurons. Use of fluorescence imaging of neurons treated with dantrolene is not technically possible because of its intense absorption at critical wavelengths, so alternative methods will be required.

In conclusion, we used optogenetic tools to study volatile anesthetic effects on presynaptic Ca^2+^ regulation and synaptic vesicle exocytosis in rodent hippocampal neurons. We identified and characterized anesthetic effects on intracellular Ca^2+^ regulation, specifically on stimulation-evoked changes in ER Ca^2+^ concentration, in hippocampal neurons from both wild-type and malignant hyperthermia-susceptible mice. We identified reduced baseline and stimulation-evoked increases in neuronal ER Ca^2+^ concentration as a novel mechanism of volatile anesthetic action. We also revealed a novel neuronal phenotype of MH susceptibility in response to the volatile anesthetics isoflurane and sevoflurane, which trigger MH. Finally, we provide functional evidence for a presynaptic role of RyR1 in synaptic transmission in the hippocampus. Taken together, our findings implicate RyR1 in presynaptic function and pave the way understanding and improving general anesthesia, and in treating those with MH-susceptibility mutations. Our results provide several important advances in elucidating the role of presynaptic ER Ca^2+^ mechanisms and RyR1 function in neuropharmacology and the actions of volatile anesthetics.

## Synthesis

Reviewing Editor: Deanna Smith, University of South Carolina Decisions are customarily a result of the Reviewing Editor and the peer reviewers coming together and discussing their recommendations until a consensus is reached. When revisions are invited, a fact-based synthesis statement explaining their decision and outlining what is needed to prepare a revision will be listed below. The following reviewer(s) agreed to reveal their identity: Isaac Pessah.

The reviewers and I agree that this is an important finding that will be of interest to eNeuro readers. A few revisions should be made. Please address the following:

1. How many separate culture days and how many individual mice were included in each culture day? Was the N reported an average of multiple neurons from a single culture or multiple cultures? This will impact whether statistical corrections used are appropriate.

2. Line 80 and Discussion should rationalize why T4826I homozygous, rather than heterozygous, animals were used as the basis for this study and how results from homozygous neurons could confound or influence more general predictions about human responses for volatile halogenated general anesthetics. In this regard, the authors may want to discuss and cite Aleman et al 2020 which reported in vivo evidence of amplified EEG depression in R163C heterozygous mice in response to anesthesia (see below).

3. Abstract Line 13: sentence as written implies that the mutation was studied in both species (rat and mouse). Actually the gene effect was only studied in the homogygous T4826I mice. Also no data is provided in this manuscript using rat neurons, only reference to pre-existing literature.

4. Methods: It is unclear how many separate culture days and how many individual mice were included in each culture day. It is unclear whether the N reported was an average of multiple neurons from a single culture or multiple cultures. As such, the broad predictive value of the statistics cannot be adequately assessed. Please clarify.

5.Lines 37, 192 and Discussion: Statement of novelty is not accurate. Aleman et al (2020) reported that MHS R163C-RYR1 heterozygous mice display significantly more pronounced EEG suppression during general anesthesia than WT mice. In fact, R163C-RYR1mice whose core body temperature were not permitted to progress to fulminant hypertheria (ie, clamped at 35oC) during anesthesia quickly transitioned to isoEEG prior to respiratory and ECG arrest. The results of study reported here should be put in context of the previous in vivo results with R16C-RYR1 mice showing significantly enhanced suppression of CNS electrical activity by a general volatile anesthetic (halothane) prior to peripheral effect and in the absence of hyperthermia and muscle rigidity. These adjustments should be made in the Significance (line 37), Results (line 193), and accommodated in the Discussion.

6. No mention of wether the the anesthetics used are know the inhibit SERCA and whether SERCA might be down regulated in this MHS line. Could altered SERCA expression be contributing to the observed effects? Finally more discussion is needed indicating the state of the science about RYR1 expression in the CNS and its pre and/or post synaptic localization.

## Author Response

Author Responses to Reviewer Comments

How many separate culture days and how many individual mice were included in each culture day? Was the N reported an average of multiple neurons from a single culture or multiple cultures? This will impact whether statistical corrections used are appropriate. Methods: It is unclear how many separate culture days and how many individual mice were included in each culture day. It is unclear whether the N reported was an average of multiple neurons from a single culture or multiple cultures. As such, the broad predictive value of the statistics cannot be adequately assessed. Please clarify.

Each culture was made up of dissociated hippocampi from 3-5 rat or mouse pups in a single culture. The N reported corresponds to the number of neurons recorded under the described conditions. Each treatment group included at least 3 cultures for each treatment group as described in lines 117-118.

Line 80 and Discussion should rationalize why T4826I homozygous, rather than heterozygous, animals were used as the basis for this study and how results from homozygous neurons could confound or influence more general predictions about human responses for volatile halogenated general anesthetics. In this regard, the authors may want to discuss and cite Aleman et al 2020 which reported in vivo evidence of amplified EEG depression in R163C heterozygous mice in response to anesthesia (see below).

Homozygous mice were used as a reproducible model of MH since 100% of homozygous T4826I RYR1 mice develop fulminant MH while only 17% of male and 0% of female heterozygous T4826I RYR1 mice developed fulminant MH [Yuen et al. 2011]. Since individual animal responses could not be recorded in vivo to confirm response to isoflurane as mice were sacrificed at P0-P1, neurons from animals exhibiting consistent MH responses were essential to our experiments. The response to these comments is clarified in lines 100-103 as well as lines 417-421.

The in vivo evidence for amplified EEG suppression in R163C mice is addressed below.

Abstract Line 13: sentence as written implies that the mutation was studied in both species (rat and mouse). Actually the gene effect was only studied in the homogygous T4826I mice. Also no data is provided in this manuscript using rat neurons, only reference to pre-existing literature.

Thank you for pointing out this ambiguity. We studied wild-type rat neurons (for example in Fig. 3,4,5, and 6) in addition to mutant and wild-type mouse neurons. The abstract clarifies this in lines 13-16.

No mention of whether the anesthetics used are know the inhibit SERCA and whether SERCA might be down regulated in this MHS line. Could altered SERCA expression be contributing to the observed effects? Finally more discussion is needed indicating the state of the science about RYR1 expression in the CNS and its pre and/or post synaptic localization.

Thank you for this comment. It is unlikely that SERCA is the major mechanism for the effects observed, as SERCA is not known to be modulated by isoflurane or to be directly affected by the T4826I-RYR1 mutation.

The precise subcellular localizations of RyR subtypes are not well-defined due to the limitations in antibody specificity used for immunocytochemistry. We have included these points of discussion in lines 376-378 and 402-406.

Lines 37, 192 and Discussion: Statement of novelty is not accurate. Aleman et al (2020) reported that MHS R163C-RYR1 heterozygous mice display significantly more pronounced EEG suppression during general anesthesia than WT mice. In fact, R163C-RYR1mice whose core body temperature were not permitted to progress to fulminant hyperthermia (ie, clamped at 35oC) during anesthesia quickly transitioned to isoEEG prior to respiratory and ECG arrest. The results of study reported here should be put in context of the previous in vivo results with R16C-RYR1 mice showing significantly enhanced suppression of CNS electrical activity by a general volatile anesthetic (halothane) prior to peripheral effect and in the absence of hyperthermia and muscle rigidity. These adjustments should be made in the Significance (line 37), Results (line 193), and accommodated in the Discussion.

Thank you for pointing out this relevant study. We have revised the manuscript to include these points in lines 47-48, 64-68 (moved from Results to Introduction for better flow), and 380-388. We appreciate this comment which enhances to our manuscript
